# Calcium Dobesilate Modulates PKCδ-NADPH Oxidase- MAPK-NF-κB Signaling Pathway to Reduce *CD14*, *TLR4*, and *MMP9* Expression during Monocyte-to-Macrophage Differentiation: Potential Therapeutic Implications for Atherosclerosis

**DOI:** 10.3390/antiox10111798

**Published:** 2021-11-11

**Authors:** Florence Njau, Hermann Haller

**Affiliations:** Division of Nephrology, Hannover Medical School, 30625 Hannover, Germany; haller.hermann@mh-hannover.de

**Keywords:** atherosclerosis, calcium dobesilate, inflammation, monocyte-macrophage differentiation, oxidative stress, PKCδ

## Abstract

Monocyte-to-macrophage differentiation results in the secretion of various inflammatory mediators and oxidative stress molecules necessary for atherosclerosis pathogenesis. Consequently, this differentiation represents a potential clinical target in atherosclerosis. Calcium dobesilate (CaD), an established vasoactive and angioprotective drug in experimental models of diabetic microvascular complications reduces oxidative stress and inhibits inflammation via diverse molecular targets; however, its effect on monocytes/macrophages is poorly understood. In this study, we investigated the anti-inflammatory mechanism of CaD during phorbol 12-myristate 13-acetate (PMA)-induced monocyte-to-macrophage differentiation in in vitro models of sepsis (LPS) and hyperglycemia, using THP-1 monocytic cell line. CaD significantly suppressed CD14, TLR4, and MMP9 expression and activity, lowering pro-inflammatory mediators, such as IL1β, TNFα, and MCP-1. The effects of CaD translated through to studies on primary human macrophages. CaD inhibited reactive oxygen species (ROS) generation, PKCδ, MAPK (ERK1/2 and p38) phosphorylation, NOX2/p47phox expression, and membrane translocation. We used hydrogen peroxide (H2O2) to mimic oxidative stress, demonstrating that CaD suppressed PKCδ activation via its ROS-scavenging properties. Taken together, we demonstrate for the first time that CaD suppresses CD14, TLR4, MMP9, and signature pro-inflammatory cytokines, in human macrophages, via the downregulation of PKCδ/NADPH oxidase/ROS/MAPK/NF-κB-dependent signaling pathways. Our data present novel mechanisms of how CaD alleviates metabolic and infectious inflammation.

## 1. Introduction

Atherosclerosis is a complex disease with poorly understood mechanisms that contribute to the disease pathogenesis but involve inflammation and oxidative stress. During its pathogenesis, circulating monocytes adhere to endothelial cells and the vessel wall and differentiate into macrophages [1,2].

In particular, monocyte-to-macrophage differentiation associates with high expression of cluster of differentiation (CD) [1,2] and Toll-like Receptor (TLR) [3,4] family members. Moreover, this process also results in the activation of enzyme systems involved in reactive oxygen species (ROS) production (i.e., leukocyte NADPH oxidase) [5], leading to oxidative stress that occurs in parallel with pro-inflammatory molecules [6] and matrix metalloproteinases [7,8] activation. All these processes contribute to atherosclerosis initiation and progression. For example, CD14 macrophages are found in complicated atherosclerotic lesions [2], and atherosclerosis progression delays when Nox2 (an essential component of NADPH oxidase) is inhibited in animal models [9,10].

Given the above-described elements, strategies to prevent monocytes infiltration and/or differentiation comprise an attractive approach to treat atherosclerosis and other related vascular disorders.

Calcium dobesilate (CaD) is a vasoactive and angioprotective drug having a unique and multi-target mode of action in several experimental studies and different animal models of diabetic microvascular complications. CaD reduces oxidative stress, inflammation, and vascular complications via diverse molecular targets [11,12,13,14]. At present, CaD is prescribed to treat microvascular damage-related diseases—diabetic retinopathy and diabetic nephropathy; however, most studies have focused only on endothelial cells.

The effect of CaD on monocytes/macrophages (the primary source of inflammatory mediators and oxidative stress) and the mechanisms of how CaD modulates monocyte-to-macrophage differentiation remain unknown. To the best of our knowledge, only three studies have addressed the CaD effects on monocytes/macrophages, including leukocyte adhesion to endothelial cells [11], protection of human peripheral mononuclear cells from oxidation [15], and apoptosis [16].

This study investigated CaD effects on monocyte-to-macrophage differentiation and its associated inflammation in PMA-, high glucose-, and LPS-treated THP-1 cells. Moreover, we validated our results in primary human M-CSF-derived macrophages. We report for the first time that CaD downregulates CD14, TLR4, MMP9, and other pro-inflammatory cytokines in THP-1 and primary human macrophages and describe the molecular mechanisms involved—PKCδ-NADPH Oxidase-ROS- MAPK-NF-κB signaling pathways.

## 2. Materials and Methods

### 2.1. Materials

THP-1 (ACC 16) human acute monocytic leukemia cells were obtained from the German Collection of Microorganisms and Cell Cultures (DSMZ) (Braunschweig, Germany). The following compounds were purchased from Sigma–Aldrich (Steinheim, Germany): phorbol 12-myristate 13-acetate (PMA; P8139), lipopolysaccharide (LPS; L2887), glucose (G-7021), calcium dobesilate (CaD; SML0488), diphenyleneiodonium chloride (DPI, a NOX2 inhibitor; D2926), SB203580 (a p38 MAPK inhibitor; S8307), Bay 11-7085 (an NF-κB inhibitor; B5681), H2DCFDA (2′,7′-dichlorofluorescin diacetate, an ROS indicator; D6883), MG132 (proteasome inhibitor; M8699), the phosphatase inhibitors sodium orthovanadate (Na3VO4; S6508), sodium fluoride (NaF; S7920), and PhosSTOP™ (4906845001), and the serine protease inhibitors PMSF (phenylmethanesulfonyl fluoride; P7626) and leupeptin (L851). The MAPK inhibitor (U0126; V1121) was from Promega, (Walldorf, Germany), while the PKCδ inhibitor rottlerin (sc-3550) and the calpain inhibitor II ALLM (CAS 136632-32-1) were purchased from Santa Cruz (Heidelberg, Germany). H_2_O_2_ was from Carl Roth GmbH, (Karlsruhe, Germany). Antibodies were purchased from Cell Signaling (Frankfurt am Main, Germany), including antibodies against p-ERK1/2 (#4377), ERK1/2 (#9102), pP38 (#4511), P38 (#9212), pPKCδ-Tyr311 (#2055), PKCδ (#2058), pIκB-α (#2859), IκB-α (#4814), CD11b (#49420), TLR2 (#12276), MMP9 (#13667), ß-Tubulin (#2146), and rabbit- (#7074) and mouse- (#7076) IgG-HRP-linked antibodies. Anti-GAPDH (sc-32233), -TLR4 (sc-293072), -pJNK (sc-6254), and -pPKCβII (sc-365463) were from Santa Cruz, (Heidelberg, Germany). BD Biosciences (Heidelberg, Germany): p47phox (#610355), R&D Systems (Wiesbaden, Germany), CD14 (MAB3832), and GeneTex (Eching, Germany), CD36 (GTX100642). ELISA kits: IL1β (88-7261-22), TNFα (88-7346-22), and MCP-1 (88-7399-22) were purchased from ThermoFisher Scientific (Darmstadt, Germany).

### 2.2. THP-1 Cells Culture, Differentiation, and Treatment

Cells were cultured in RPMI-1640 (P04-18047; PAN Biotech) supplemented with 20% fetal bovine serum (FBS), penicillin (100 U/mL), and streptomycin (100 μg/mL), and incubated at 37 °C in a humidified atmosphere of 5% CO_2_. For all experiments, THP-1 cells were seeded at a density of 1 × 10^6^/mL and treated for various time points with PMA, LPS, high glucose, or H_2_O_2_. In preliminary studies, we established nontoxic but optimal concentrations and time points for the compounds used in the study (data not shown). On the day of treatment, we incubated the cells for 1 h with the following pharmacological agents: CaD, DPI (1 µM) [17], SB203580 (5 µM) [18], U0126 (10 µM) [18], rottlerin (250 nM or 2 µM) [18], Bay 11-7085 (5 µM) [19], MG132 (10 µM) [20], or ALLM (10 µM) [21]. We then stimulated the cells with 30 nM PMA [22], 100 ng/mL LPS [23], 25 mM glucose [24], or 10 mM H_2_O_2_ [25], for varying time periods. In each case, we compared the inhibitors with their vehicle control to rule out nonspecific cytotoxicity.

### 2.3. Primary Monocyte Differentiation and Treatment

Human peripheral blood mononuclear cells (PBMCs) were isolated from buffy coats (German Red Cross: DRK-Blutspendedienst NSTOB, Springe, Germany) by ficoll gradient centrifugation. According to the supplier’s protocol, we purified monocytes from PBMCs using CD14+ beads (Miltenyi Biotech, Bergisch Gladbach, Germany). We cultured the purified monocytes in RPMI-1640 as THP-1cells but with 10% FBS for 1 h to allow recovery from stress, and then treated the monocytes with CaD (2.5–10 µM) for 1 h before inducing differentiation. To induce differentiation, we treated the monocytes with growth factor M-CSF (50 ng/mL) (Peprotech, NJ, USA) for 5 days at 37 °C, 5% CO_2_. The medium was changed on day 3 during this period. On day 5, cells were analyzed by immunoblotting or treated with 100 ng/mL LPS for 24 h for cytokine production analysis.

### 2.4. Assays of Cell Viability and Adhesion

We first used a CCK-8 kit (Dojindo Molecular Technologies, Munich, Germany) to test the effect of CaD alone on cellular viability. Briefly, THP1 cells were seeded into 96-well plates, in triplicate, and treated with various concentrations of CaD (0, 2.5, 5, 10, and 20 µM) for 24, 48, and 72 h before the addition of CCK-8 solution. We then used a Tecan Microplate Reader (Genios; Männedorf, Switzerland) to measure optical density at 450 nm. Since no toxic effects were detected at all concentrations, subsequent experiments were conducted using CaD concentrations lower than 20 μM.

We next investigated cell adhesion by crystal violet staining and bright field microscopy. For crystal violet staining, THP-1 cells were treated for various time points with 30 nM PMA in the presence of 0–10 µM CaD. Non-adherent cells were removed by three gentle rinses with PBS and adherent cells fixed with 4% paraformaldehyde, washed twice with demineralized water, and 0.1% crystal violet stain added and incubated at room temperature for 20 min. Stained cells were left to air-dry and eluted with 10% acetic acid. Absorbance was measured as the optical density (ODs) at 595 nm, using a Tecan microplate reader. For qualitative analysis of monocyte/ macrophage adhesion, phase-contrast images were taken using bright-field microscopy after 48-h stimulation by PMA.

### 2.5. Quantitative RT-PCR Analysis

Total RNA was isolated using RNeasy miniprep kits from Qiagen, Hilden, Germany) and qRT-PCR performed by a LightCycler 96 Real-Time PCR System using Taqman RT-PCR with the following Applied Biosystems primers from Dreieich, Germany: PKCα (Hs00176973_m1), PKCß (Hs00176998_m1), PKCδ (Hs01090047_m1), NOX2 (Hs00166163_m1), MMP9 (Hs00957562_m1), MCP-1/CCL2 (Hs00234140_m1), IL1β (Hs00174097_m1), TNFα (Hs00174128_m1), TLR4 (Hs00152939_m1), TLR2 (Hs00610101_m1), CD14 (Hs02621496_s1), CD36 (Hs00354519_m1). Quantification was carried out using LightCycler 96 software (Roche Diagnostics, Mannheim, Germany), and the amount of RNA was expressed as the expression ratio relative to the housekeeping gene *GUSB* (Hs00939627_m1). Quantitative PCR for CD11b (forward primer 5′-CAGCCTTTGACCTTATGT-3′ and reverse primer 5′-CCTGTGCTGTAGTCGCA-3′) and the housekeeping gene Actin B (KSPQ12012, Sigma–Aldrich, Steinheim, Germany) was performed using SBYR Green RT-PCR (Applied Biosystems, Dreieich, Germany).

### 2.6. Cytokine Production

We used PMA to differentiate THP-1 cells to macrophages in the presence of CaD for 48 h or M-CSF to differentiate human blood monocytes to macrophages for 5 days. PMA or M-CSF-derived macrophages were then stimulated with 100 ng/mL LPS. Supernatants from three independent experiments were collected after 24 h and pooled, and IL1β, TNFα, and MCP-1 levels were measured by ELISA, according to the manufacturer’s instructions. The cells were lysed for cellular protein content measurement as described below for immunoblotting. Standard curves were then generated by plotting the pg/mL concentrations versus absorbance values of the standards and used to quantify the levels of cytokines released by the cells. Detection limits were 2 pg/mL, 4 pg/mL, and 7 pg/mL for IL1β, TNFα, and MCP-1, respectively. Final cytokine concentrations (pg/mL) were normalized to the protein concentration per ml of each sample and displayed as pg/mg of protein in the figures.

### 2.7. Determination of Intracellular ROS

Intracellular reactive oxygen species (ROS) generation was measured by using the cell-permeable indicator H_2_DCFDA (Sigma-Aldrich, Steinheim, Germany). Briefly, THP-1 cells were loaded with 5 µM H_2_DCFDA for 60 min, washed with PBS washed, and pretreated with various CaD concentrations before stimulation with PMA for different durations. ROS-dependent fluorescence was measured by a microplate reader at excitation 485 nm and emission 535 nm.

### 2.8. MMP9 Gelatin Zymography

To determine MMP9 enzymatic activity by gelatin zymography, THP-1 cells were treated for 24 h in a serum-free medium, and 10 μL of cell-free conditioned media was collected by centrifugation. The supernatant was then mixed with nonreducing Laemmli sample buffer and 10 μL of the mixture was subjected to gel electrophoresis in 10% SDS-PAGE containing 0.1% (*w*/*v*) gelatin. Gels were processed as previously described [26]. The gelatinolytic activity was normalized to the total protein content of the cultured cells.

### 2.9. Immunoblotting

Whole-cell lysates were prepared in complete lysis buffer (RIPA) containing the following inhibitors of proteolysis and dephosphorylation: 1 mM PMSF, 1 mg/mL aprotinin, 1 mg/mL leupeptin, 1 mM Na3VO4, 1 mM NaF, PhosSTOP™ (4906845001; Sigma–Aldrich, Steinheim, Germany). Following incubation for 10 min at 4 °C and centrifugation (13,000× *g*, 10 min), cells were treated with lysis buffer without detergents, followed by three freeze- (−80 °C for 1 h) thaw cycles. After another centrifugation (13,000× *g*, 25 min), soluble cytosolic fractions were removed, and the remaining pellets were treated with complete lysis buffer and sonicated for 15 s. The samples were again centrifuged for 10 min at 4 °C, and the soluble supernatant containing the membrane fraction was removed and saved.

We next subjected proteins to gel electrophoresis through 10–12.5% polyacrylamide, and electro transferred them to polyvinylidene difluoride membranes (MerckMillipore, Darmstadt, Germany) Burlington, MA, USA). Membranes were blocked with 3% BSA for 1 h at room temperature and incubated overnight, at 4 °C, with 1:1000 dilution of each of the following primary antibodies: p-ERK1/2, ERK1/2, pP38, P38, PKCδ, pIκB-α, IκB-α, CD11b, TLR2, MMP9, and 1:500 dilution of GAPDH, p47phox, TLR4, pJNK, and pPKCβII, CD14, and CD36. Secondary antibodies (1:2000), conjugated to horseradish peroxidase, were used to detect protein bands using an enhanced chemiluminescence (ECL) method. Bands were normalized to GAPDH or β-tubulin as internal controls. The expression of phosphorylated proteins was normalized to total protein levels.

### 2.10. Statistical Analysis

Data are expressed as means ± SEMs. The significance of differences between groups was examined using either a Student’s *t*-test or one-way ANOVA, as appropriate. *p* < 0.05 was considered statistically significant.

## 3. Results 

### 3.1. Calcium Dobesilate (CaD) Inhibits CD14 and TLR4 Expression during Monocyte-to-Macrophage Differentiation

PMA-induced THP-1 cell differentiation is a well-accepted in vitro model for studying monocyte-to-macrophage differentiation [27]. Consequently, we first determined the cytotoxic effects of CaD for 24, 48, and 72 h on THP-1 cells, using CCK-8 cell viability assays, showing that CaD did not cause any detectable cell death in monocytes at the indicated concentrations (Supplementary Figure S1A).

Since PMA treatment induces greater differentiation of THP-1 cells (reflected by the increased adherence and expression of surface markers associated with macrophage differentiation [27]), we studied the effect(s) of CaD on PMA-induced monocyte-to-macrophage differentiation. We treated THP-1 cells with 30 nM PMA for various time points, in the presence or absence of CaD (0–10 µM), demonstrating that CaD did not inhibit PMA-induced THP-1 cell adhesion (Supplementary Figure S1B,C).

**Figure 1:**
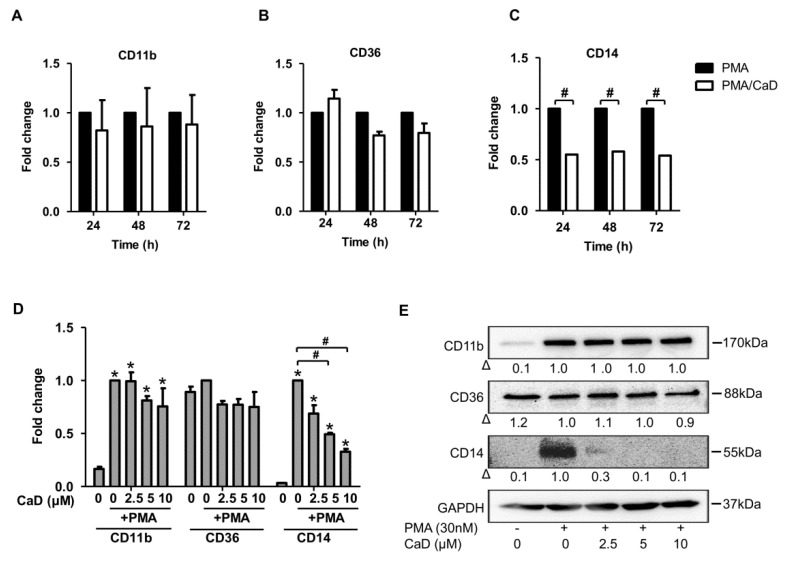
Calcium dobesilate (CaD) inhibits PMA-induced CD14 expression. THP-1 monocytes were pretreated with 10 µmol/L CaD for 1 h, followed by stimulation with PMA (30 nmol/L) for various time points (**A**–**C**), or treated with various concentrations (0–10 µmol/L) of CaD for 1 h (**D**,**E**) followed by PMA treatment for 72 h. Transcript levels of the indicated genes were measured by quantitative RT-PCR (**A**–**D**), and protein levels measured by Western blotting (**E**). Δ, fold-change normalized to PMA only (*n* = 3–4, mean ± SEM. * *p* < 0.05 vs. no treatment, ^#^ *p* < 0.05 vs. PMA only, Student’s *t*-test (**A**–**C**), or one-way ANOVA (**D**)). The Western blot represents one from at least three independent experiments.

**Figure 2:**
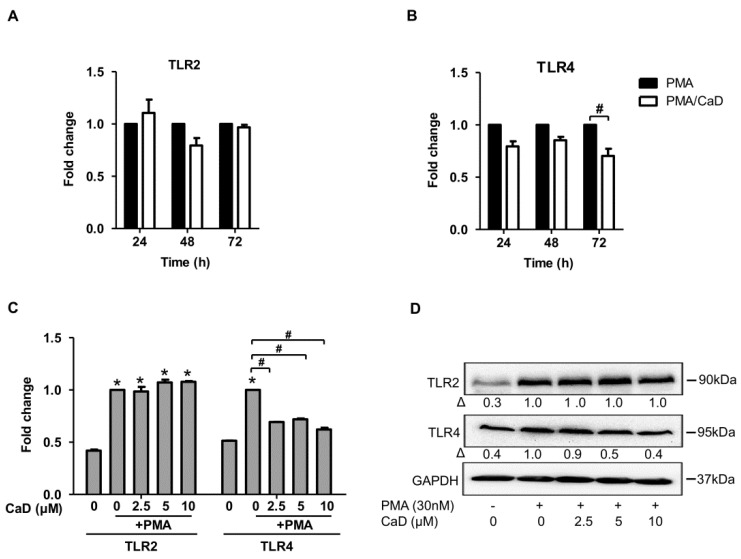
CaD on TLR4 expression during monocyte-to-macrophage differentiation. THP-1 monocytes were pretreated with 10 µmol/L of CaD for 1 h followed by stimulation with PMA (30 nmol/L) for various time points (**A**,**B**), or THP-1 monocytes were treated with various concentrations (0–10 µmol/L) of CaD for 1 h (**C**–**D**) followed by PMA treatment for 72 h. TLR2 and TLR4 transcripts (**A**–**C**) and protein levels (**D**) were measured by quantitative RT-PCR and Western blotting, respectively. Δ, fold-change normalized to PMA only (*n* = 3–4, mean ± SEM. * *p* < 0.05 vs. no treatment, ^#^ *p* < 0.05 vs. PMA only, Student’s *t*-test (**A**,**B**), or one-way ANOVA (**C**)). The Western blot represents one from at least three independent experiments.

One feature of monocyte-to-macrophage differentiation is the loss or gain of expression of an array of genes/proteins. Consequently, we analyzed the effect of CaD on the expression of molecular markers of macrophage differentiation, namely CD11b, CD14, CD36, TLR2, and TLR4. As expected, PMA increased CD36, CD11b, and CD14 expression (Figure 1A–C). Although CaD did not affect CD36 and CD11b expression during PMA-induced differentiation, it significantly decreased CD14 expression in a time-independent (Figure 1C) and dose-dependent manner (Figure 1D,E). Moreover, CaD significantly downregulated TLR4 expression at 72 h treatment but not TLR2 (Figure 2A,B). CaD dose-dependently downregulated TLR4, but not TLR2, expression (Figure 2C,D). These results suggest that CaD selectively inhibits certain aspects of monocyte-to-macrophage differentiation and inflammation. Elevated expression and activity of CD14, TLR2, and TLR4 correlate with advanced atherosclerotic lesions, followed by plaque rupture and myocardial infarction [2,3,28]. Hence, we speculate that CaD may alter or improve some aspects of atherosclerosis, although further studies are required to elaborate on this point.

### 3.2. CaD Inhibits Inflammation during Monocyte-to-Macrophage Differentiation

**Figure 3:**
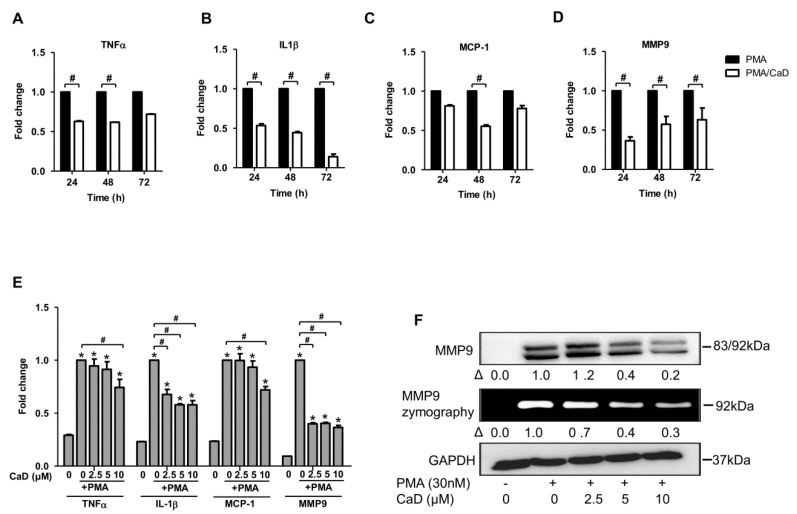
CaD inhibits PMA-induced inflammation during PMA-induced monocyte-to-macrophage differentiation. THP-1 monocytes were pretreated with CaD (10 µmol/L) for 1 h followed by stimulation with PMA (30 nmol/L) for various time points (**A**–**D**), or THP-1 monocytes were treated with various CaD concentrations (0–10 µmol/L), followed by PMA treatment for 48 h (**E**,**F**). TNFα, IL-1β, MCP-1, and MMP9 transcript levels were measured by quantitative RT-PCR, and MMP9 protein levels and activity were measured by Western blotting and gelatin zymography, respectively. Δ, fold-change normalized to PMA only (*n* = 3–4, mean ± SEM. * *p* < 0.05 vs. no treatment, ^#^ *p* < 0.05 vs. PMA only, Student’s *t*-test (**A**–**D**), or one-way ANOVA (**E**)). The Western blots represent one from at least three independent experiments.

Besides monocyte adhesion and expression of common surface markers during monocyte-to-macrophage differentiation, various inflammatory cascades are evoked during the differentiation process. CD14 and TLR4 facilitate detection of bacterial lipopolysaccharide (LPS); consequently, CD14 and TLR4 knockout mouse macrophages have impaired NF-κB pathway activation, resulting in deficient LPS-induced IL6 and TNFα production [29,30,31]. Hence, CaD-mediated CD14 and TLR4 inhibition in THP-1 macrophages could explain why CaD inhibited NF-κB activation and the release of its downstream products—IL1β, IL6, and TNFα, in LPS-treated rat models of systemic inflammation [32]. In this study, we investigated the effect of CaD on TNFα, IL1β, and MCP-1 expression during PMA induced monocyte-to-macrophage differentiation; CaD significantly decreased the expression of these cytokines, the optimal time point for all cytokines being 48 h (Figure 3A–D). CaD dose-dependently decreased IL1β expression, but 10 µM was the most effective concentration that inhibited all the pro-inflammatory cytokines expression (Figure 3E,F). 

Macrophage-derived matrix metalloproteases (MMPs) are highly expressed in atherosclerotic plaques and implicated in plaque rupture. Because MMP-9 is one of the key regulators of vascular complications, we studied the effect of PMA on MMP9 expression and activity during monocyte-to-macrophage differentiation. Treatment with CaD inhibited MMP9 expression time-independently (Figure 3D) and dose-dependently (Figure 3E,F). Gelatin zymography is widely used to study gelatinase activation; however, this technique has some pitfalls due to the complexity of the activation process and the inherent limitations of SDS-PAGE, which make identification of active species by gelatin zymography not straightforward, especially if one cannot achieve a good band separation. This study could not delineate the inactive 92 kDa and the active 83 kDa species by gelatin zymography (Figure 3F). However, we demonstrated the presence of both MMP9 species by immunoblotting. Additionally, unlike other MMPs, which are constitutively expressed, MMP9 tends to be inducible by growth factors and inflammatory stimuli; thus, CaD treatment inhibited MMP9 expression and production. Given the importance of inflammation in atherosclerosis pathogenesis, our data suggest CaD as a promising inhibitor of atherosclerosis development and progression.

### 3.3. The Effect of CaD Is Not Limited to PMA-Induced Monocyte-to-Macrophage Differentiation and Inflammation: Effects of LPS and High Glucose Stimulation

Based on the observation that CaD downregulated CD14 and TLR4 expression during monocyte-to-macrophage differentiation and that CD14 and TLR4 are important in detecting bacterial LPS, we asked whether CaD could inhibit LPS-induced CD14 and TLR4 expression in THP-1 cells. As depicted in Supplementary Figure S2A,B, CaD inhibited LPS-induced CD14 (mRNA), TLR4, and MMP9 expression. We did not detect CD14 protein expression by immunoblotting, possibly because THP-1 cells express low CD14 levels [27].

**Figure 4:**
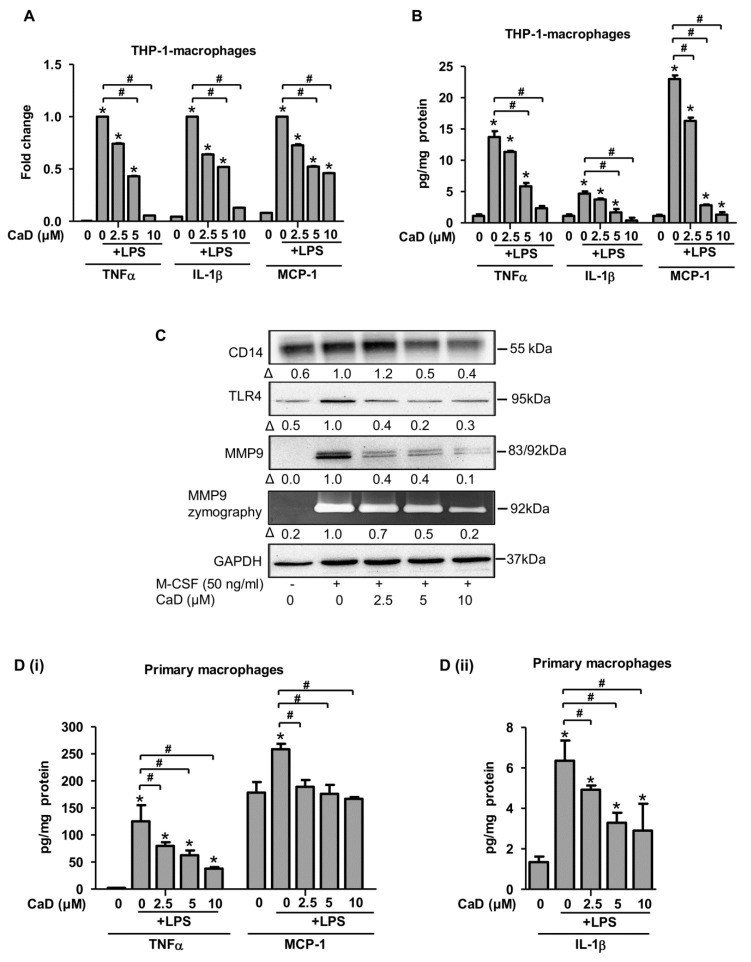
CaD inhibits primary human monocyte-to-macrophage differentiation and LPS-induced inflammation. Primary human monocytes were treated with various CaD concentrations (0–10 µmol/L) for 1 h followed by M-CSF (50 ng/mL) treatment for 5 days (**A**). Protein levels and MMP9 activity were measured by Western blotting and gelatin zymography, respectively. THP-1 cells and primary human monocytes were differentiated into macrophages in the presence of various CaD concentrations (0–10 µmol/L) for 48 h and 5 days, respectively (**B**–**D**). Macrophages were then stimulated with LPS (100 ng/mL) for 24 h. TNFα, IL1β, and MCP-1 transcript levels (**B**) were measured by quantitative RT-PCR, and protein levels (**C**,**D**) in the conditioned media were measured by ELISA (**B**). Δ, fold-change normalized to PMA only (*n* = 3, mean ± SEM. * *p* < 0.05 vs. no LPS, ^#^ *p* < 0.05 vs. LPS only, one-way ANOVA).

Since THP-1 cells express low CD14 levels, they are less responsive to LPS treatment [27]. To investigate the effect of LPS and CaD on the expression of inflammatory molecules, we differentiated THP-1 cells to macrophages with PMA in the presence of CaD for 48 h, treated the macrophages with LPS for 24 h, and measured the expression of representative pro-inflammatory molecules by ELISA and quantitative RT-PCR. Macrophages treated with LPS in the presence of CaD expressed significantly lower IL1β, TNFα, and MCP-1 (Figure 4B,C), in agreement with a previous study where CaD reduced pro-inflammatory cytokine production in a rat model of sepsis [32].

In a previous study, a herbal extract named Baihu decoction, which has hypoglycemic and antioxidant effects, significantly inhibited a CD14/TLR4/NF-kB pathway, and its associated inflammation, in a type 2 diabetic mouse model [33]. We hypothesized that based on its effect on CD14 and TLR4 expression, CaD could exert a similar mechanism in diabetic models. As illustrated in Supplementary Figure S3, CaD downregulated the expression of CD14 and TLR4, as well as MMP9, IL1β, TNFα, and MCP-1, in THP-1 cells cultured in high glucose for 48 h (to mimic chronic hyperglycemia).

### 3.4. CaD Modulates Primary Human Monocyte-to-Macrophage Differentiation and Inflammation

Although THP-1 cells are a human cell line, they are immortalized monocytic leukemia. So we looked to translate our results to primary human macrophages, using in vitro differentiation of monocytes. Specifically, isolated human monocytes were cultured with M-CSF (50 ng/mL) and various CaD concentrations. CaD significantly inhibited CD14, TLR4, and MMP9 protein expression than the M-CSF only treated macrophages in a concentration-dependent manner (Figure 4A). Finally, we wanted to study the effects of CaD on CD14 and TLR4 inflammatory signaling for the differentiated primary macrophages when stimulated with LPS (100 ng/mL) for 24 h. As a readout, we measured the levels of pro-inflammatory cytokines (IL-1β, TNFα, and MCP-1). In agreement with the decreased CD14 and TLR4 expression due to CaD, we observed a significant reduction in all the cytokines tested in response to LPS (Figure 4D). Therefore, the effects of CaD during PMA-induced THP-1 differentiation translate through to human primary macrophage function.

### 3.5. Signaling Pathways Affected by CaD during Monocyte-to-Macrophage Differentiation

**Figure 5:**
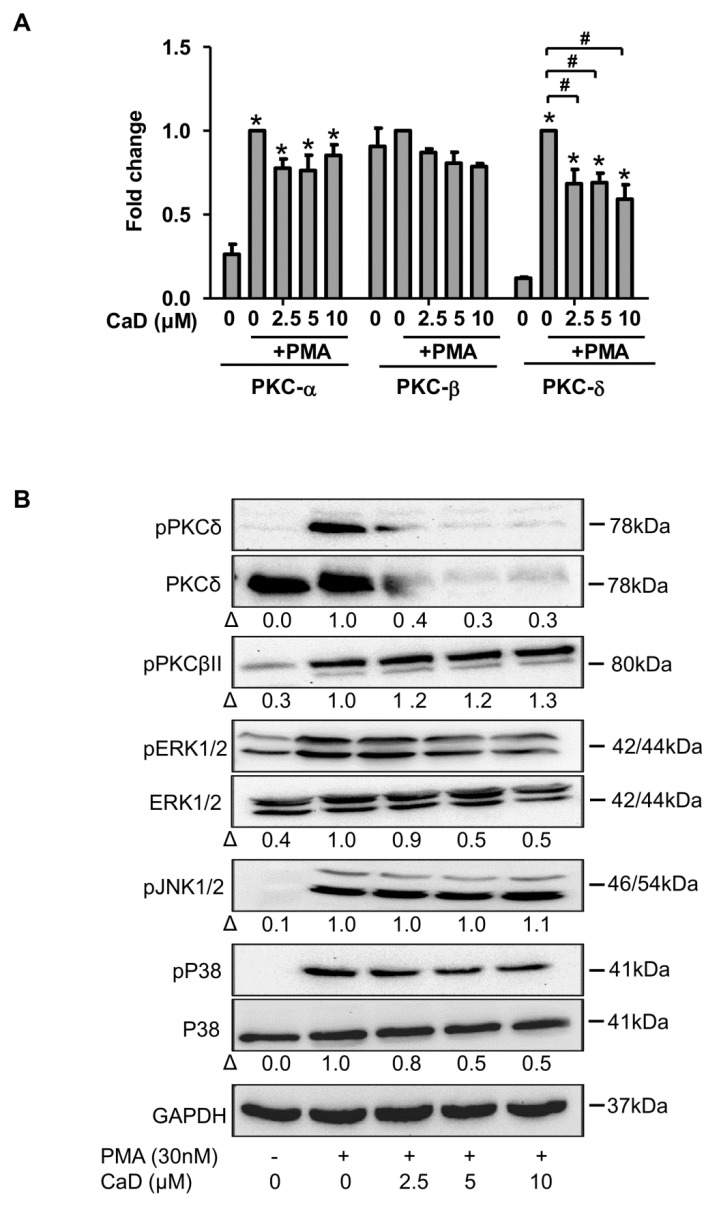
Effect of CaD on PKCδ-MAPK signaling during PMA-induced monocyte-to-macrophage differentiation. THP-1 monocytes were pretreated with various concentrations (0–10 µmol/L) of CaD for 1 h, followed by PMA treatment for 24 h (**A**) or 30 min (**B**). PKCα, PKCßII, and PKCδ transcript levels (**A**) were measured by quantitative RT-PCR, and phosphorylation of PKC and MAPK pathway components (**B**) was measured by Western blotting. Δ, fold-change normalized to PMA only (*n* = 3, mean ± SEM. * *p* < 0.05 vs. no treatment, ^#^ *p* < 0.05 vs. PMA only, one-way ANOVA). The Western blots represent one from at least three independent experiments.

We next sought to determine the molecular mechanisms by which CaD inhibits monocyte-to-macrophage differentiation and inflammation, especially as it is dependent on the protein kinase C and MAPK pathways because PMA is a potent PKC activator and MAPK is downstream of PKC [34]. PMA treatment significantly upregulated PKCδ expression but weakly increased PKCα and PKCβII at the gene level (Figure 5A). Moreover, PMA significantly increased PKCδ and pPKCβII phosphorylation (Figure 5B). We did not detect PKCα protein by immunoblotting, likely due to its low expression in THP-1 cells [27]. THP-1 cells treatment with CaD, during differentiation, did not affect PKCα and PPKCβII expression, but CaD significantly reduced PKCδ activation (Figure 5A,B), suggesting that CaD specifically inhibits the PKCδ-dependent signaling pathway during PMA-induced monocyte-to-macrophage differentiation.

Furthermore, CaD inhibited ERK1/2 and p38 phosphorylation induced by PMA treatment (Figure 5B), but not JNK1/2 phosphorylation. These results suggest that CaD preferentially inhibits ERK1/2 and P38-related pathways.

In addition to PMA, LPS and high glucose are known to activate PKCδ and MAPK [35,36,37,38]. Accordingly, we investigated the effect of CaD under LPS and high glucose treatment of THP1 cells, finding that CaD significantly downregulated the activation of PKCδ, ERK1/2, and p38 (Supplementary Figure S4A,B). However, we did not detect phosphorylation of PKCδ by immunoblotting (even at time points ranging from 5 min to 48 h), possibly because LPS and high glucose more weakly induce PKC phosphorylation than PMA. 

### 3.6. CaD Inhibits Monocyte-to-Macrophage Differentiation and Inflammation via PKCδ, MAPK Pathway

**Figure 6:**
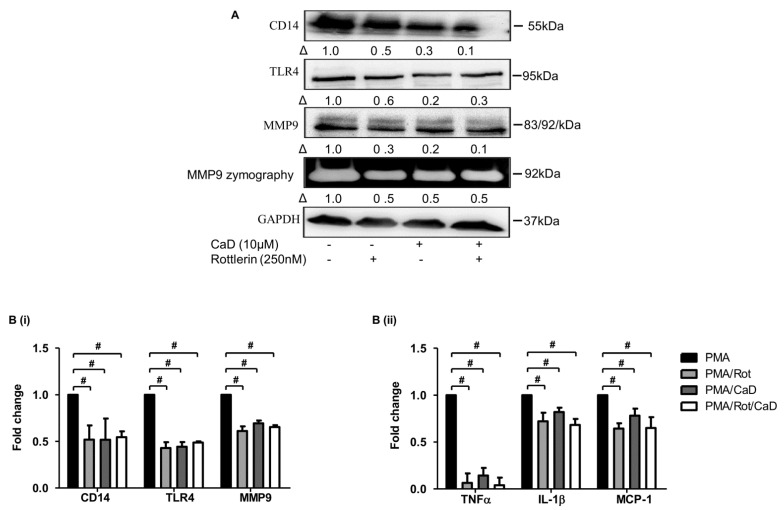
Inhibition of monocyte-to-macrophage differentiation and inflammation by CaD involves PKCδ-dependent signaling. THP-1 monocytes were pretreated with the PKCδ inhibitor rottlerin (Rot) for 1 h, followed by CaD for 1 h, and then treated with PMA (30 nmol/L) for 72 h. The differentiation and inflammation marker expression were measured by Western blotting (**A**), MMP9 activity measured by gelatin zymography, and transcript levels (**B**) measured by quantitative RT-PCR. Δ, fold-change normalized to PMA only (*n* = 3, mean ± SEM. ^#^ *p* < 0.05 vs. PMA only, one-way ANOVA). The Western blot represents one from at least three independent experiments.

To determine whether the effect of CaD on PKCδ is necessary to inhibit CD14, TLR4, MMP9, and pro-inflammatory cytokine expression, we inhibited PKCδ (rottlerin) in the PMA-THP-1 model. Figure 6 indicates that, compared to rottlerin, CaD likewise significantly downregulated CD14, TLR4, MMP9, TNFα, IL1β, and MCP-1 expression, suggesting that CaD inhibits monocyte-to-macrophage differentiation by inhibiting PKCδ activation.

**Figure 7:**
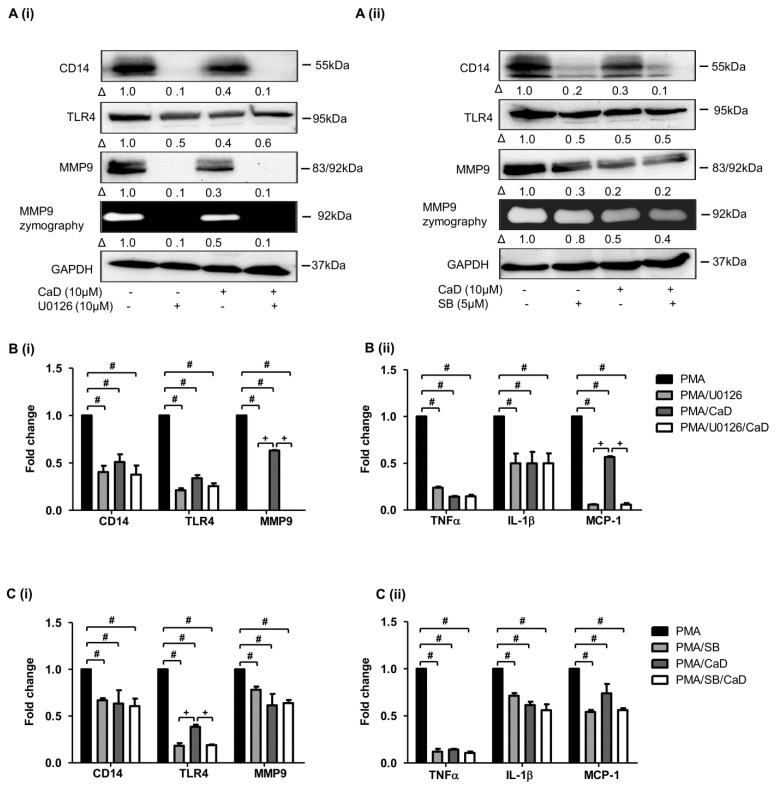
CaD suppresses the MAPK signaling to abolish monocyte-to-macrophage differentiation and inflammation. THP-1 monocytes were pretreated with an ERK1/2 (U0126) or P38 inhibitor (SB203580) for 1 h, followed by CaD for 1 h, and then treated with PMA (30 nmol/L) for 72 h. The differentiation and inflammation markers expression were measured by Western blotting (**A**), MMP9 activity was measured by gelatin zymography, and transcript levels (**B**,**C**) were measured by quantitative RT-PCR. Δ, fold-change, normalized to PMA only (*n* = 3, mean ± SEM. ^+^ *p* < 0.05 vs. inhibitor treatment, ^#^ *p* < 0.05 vs. PMA only, one-way ANOVA). The Western blots represent one from at least three independent experiments.

Moreover, inhibiting MAPK downregulates CD14, TLR4, MMP9, and inflammatory marker expression [39,40,41]; we, therefore, inhibited ERK1/2 (U0126) and p38 (SB203580) in the PMA THP-1 cell model in the presence or absence of CaD. Like CaD treatment, both MAPK inhibitors significantly inhibited CD14 and TLR4, MMP9, IL1β, TNFα, and MCP-1 expression (Figure 7). Put together, our findings indicate that PKCδ, ERK1/2, and p38 are the key pathways that CaD targets to inhibit monocyte-to-macrophage differentiation and inflammation.

### 3.7. NF-kB Is Involved in CaD-Mediated Inhibition of Monocyte-to-Macrophage Differentiation and Inflammation

**Figure 8:**
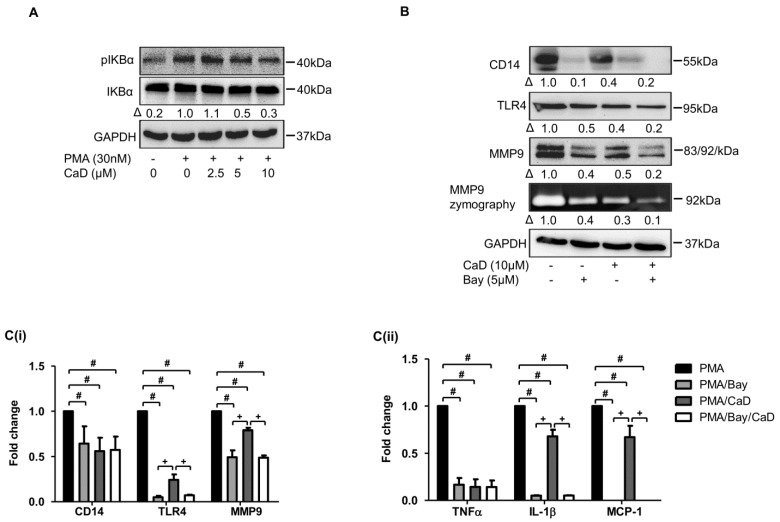
CaD inhibits monocyte-to-macrophage differentiation and inflammation in an NF-κB-dependent manner. THP-1 monocytes were pretreated with various concentrations (0–10 µmol/L) of CaD for 1 h, followed by PMA treatment for 10 min (**A**). THP-1 monocytes were pretreated with an NF-κB inhibitor (Bay 11-7085) for 1 h, followed by CaD for 1 h, and then treated with PMA (30 nmol/L) for 72 h (**B**,**C**). IKBα phosphorylation and the differentiation and inflammation marker expression (**A**,**B**) were measured by Western blotting, MMP9 activity was measured by gelatin zymography, and transcript (**C**) levels were measured by quantitative RT-PCR. Δ, fold change normalized to PMA only (*n* = 3, mean ± SEM. ^+^ *p* < 0.05 vs. Bay treatment, ^#^ *p* < 0.05 vs. PMA only, one-way ANOVA). The Western blots represent one from at least three independent experiments.

We attempted to clarify whether CaD inhibits NF-κB expression in PMA-induced monocyte-to-macrophage differentiation and inflammation because PKC regulates NF-kB activation in macrophages [35]. Figure 8A demonstrates that CaD inhibits IκBα phosphorylation during monocyte-to-macrophage differentiation; furthermore, CaD inhibited IκBα phosphorylation in THP-1 cells treated with LPS and high glucose (Supplementary Figure S4A,B). 

By using an NF-κB inhibitor (Bay-117085), we showed that similar to CaD treatment, Bay-117085 attenuated PMA-induced expression of differentiation and inflammation markers (Figure 8B,C). These results tie well with previous studies where CaD suppressed NF-κB activation [11] and IκB-α phosphorylation [42] in in vivo and in vitro diabetic models, subsequently inhibiting inflammation.

### 3.8. Effect of CaD on NADPH Oxidase Activation

**Figure 9:**
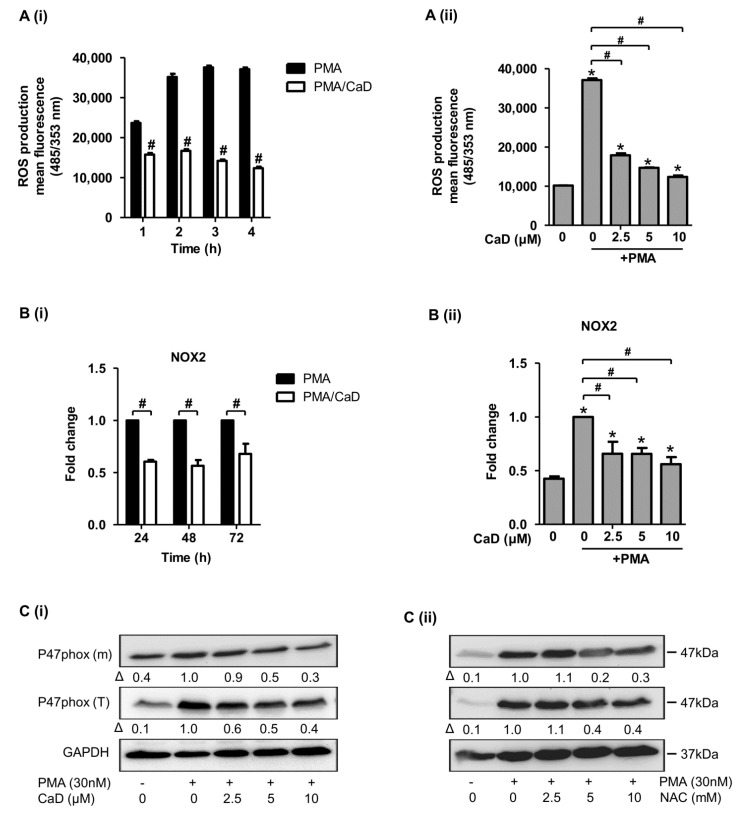
CaD modulates the ROS-NADPH oxidase pathway during monocyte-to-macrophage differentiation and inflammation. THP-1 monocytes were pretreated with CaD (10 µmol/L), or various concentrations (0–10 µmol/L) of CaD/N-acetylcysteine (NAC) for 1 h, followed by PMA (30 nmol/L) treatment for various time points (**Ai**,**Bi**), for 4 h (**Aii**), or 24 h (**Bii**,**C**). ROS production (**A**) was measured using the cell-permeable indicator H_2_DCF-DA as described in the Materials and Methods section. *NOX2* transcript levels (**B**) were measured by quantitative RT-PCR, and total p47phox protein expression (T) and membrane translocation (m) (**C**) were measured by Western blotting. Δ, fold-change normalized to PMA only (*n* = 3, mean ± SEM. * *p* < 0.05 vs. no treatment, ^#^ *p* < 0.05 vs. PMA only, Student’s *t*-test (**Ai**,**Bi**), or one-way ANOVA (**Aii**,**Bii**). The Western blots represent one from at least three independent experiments.

NADPH oxidase is critical for ROS generation in phagocytic cells, and ROS are implicated in various cell signaling processes, including monocyte-to-macrophage differentiation [43]. We next investigated the effect of CaD on PMA-triggered ROS generation, NOX-2/p47phox activation, and p47phox membrane translocation. We used N-acetylcysteine (NAC) as a ROS scavenger control to investigate its effect on p47phox activation and membrane translocation. Figure 9(Ai) shows that although ROS production increased with time, CaD quenched PMA-induced ROS production time independently; moreover, CaD blocked ROS generation dose-dependently. Both CaD and NAC inhibited PMA-triggered NOX2/p47phox expression and membrane translocation (Figure 9B,C). 

**Figure 10:**
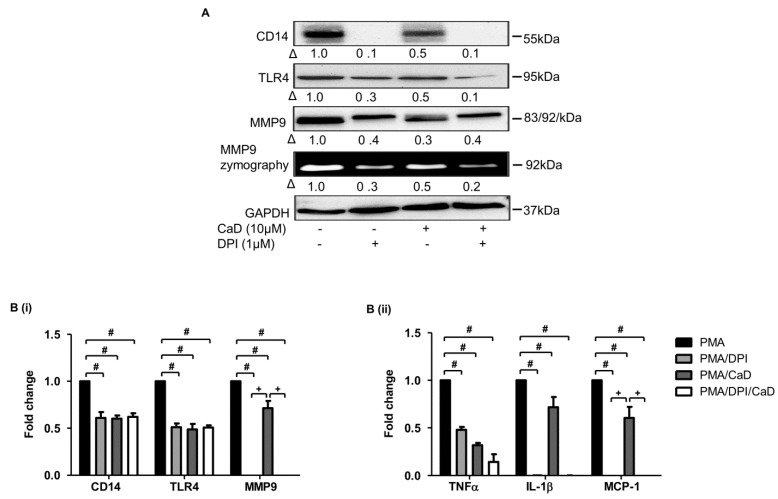
A NOX2 inhibitor (DPI) abrogates PMA-induced monocyte-to-macrophage differentiation and inflammation. THP-1 monocytes were pretreated with DPI for 1 h, followed by CaD for 1 h, and then treated with PMA (30 nmol/L) for 72 h. Differentiation and inflammation marker expression was measured by Western blotting (**A**), MMP9 activity was measured by gelatin zymography, and transcript (**B**) levels (**B**) were measured by quantitative RT-PCR. Δ, fold-change normalized to PMA only. (*n* = 3, mean ± SEM. ^+^ *p* < 0.05 vs. DPI treatment, ^#^ *p* < 0.05 vs. PMA only, one-way ANOVA). The Western blots represent one from at least three independent experiments.

ROS regulate LPS and glucose-mediated NF-kB activation and inflammatory marker expression in human monocytes/macrophages [38,44]. Therefore, we investigated the effects of CaD on LPS and high glucose-induced p47phox activation and membrane translocation. Supplementary Figure S4A,B indicates that CaD significantly inhibited LPS and glucose-induced p47phox activation and membrane translocation. We further validated the role of ROS, NADPH oxidase, and the effect of CaD during monocyte-to-macrophage differentiation and inflammation in THP-1 cells by inhibiting NADPH oxidase (NOX2, p47phox) with a NADPH oxidase inhibitor (diphenyleneiodonium chloride, DPI), or NAC, in the presence or absence of CaD. DPI suppressed CD14, TLR4, MMP9, TNFα, IL1β, and MCP-1 expression (Figure 10), and NAC suppressed CD14, TLR4, and MMP9 expression (Supplementary Figure S5A) (we did not investigate the effect of NAC on pro-inflammatory cytokines as this question has been widely discussed).

### 3.9. Proximal Signaling Mediator under CaD Treatment

After identifying signaling pathways inhibited by CaD, we used the PMA-THP-1 cell model to determine a proximal signaling mediator via the use of specific pharmacological inhibitors.

First, because PKCδ is upstream to p47phox, p38, ERK1/2, and NF-κB [45,46], we examined the effect of PKCδ on p47phox activation and membrane translocation, and ERK1/2, P38, and IKBα phosphorylation. PKCδ inhibitor (rottlerin) significantly decreased p47phox activation (T) and membrane (m) translocation. Rottlerin also decreased ERK1/2, P38, and IKBα phosphorylation in a manner comparable to CaD treatment (Supplementary Figure S6). The findings indicated that NOX2/p47phox, MAPK, and NF-κB are downstream targets of PKCδ and that CaD inhibits their activation by modulating PKCδ.

Second, we treated cells with ERK1/2, p38, and NOX2/p47phox inhibitors and challenged these with PMA in the presence or absence of CaD. PKCδ expression and activation did not change with p38, ERK1/2, or NOX2/p47phox inhibitors (data not shown); thus, we confirmed the above findings that MAPK and NOX2/p47phox are downstream of PKCδ. 

### 3.10. CaD Inhibits PKCδ via Its Antioxidant Activity

Next, we asked how CaD regulates PKCδ during monocyte-to-macrophage differentiation. It is known that PMA activates, subsequently degrades, and depletes phorbol ester-responsive PKC isoforms, such as PKCδ [47]**,** either via the ubiquitin-proteasome system [48] or Ca2^+^-activated neutral proteases, such as calpains [49]. By using MG132 (proteasome inhibitor) and ALLM (calpain inhibitor), we ruled out CaD downregulation of PKCδ via proteasome and calpain mechanisms (data not shown).

ROS is not only a downstream but also an upstream signaling molecule to PKC [38,44]. Because of our findings that CaD suppressed PKCδ activation, we hypothesized that CaD downregulates PKCδ by inhibiting ROS activity. Indeed NAC significantly inhibited PMA-induced PKCδ activation and expression (Supplementary Figure S5B), indicating that ROS acts upstream of PKCδ. 

**Figure 11:**
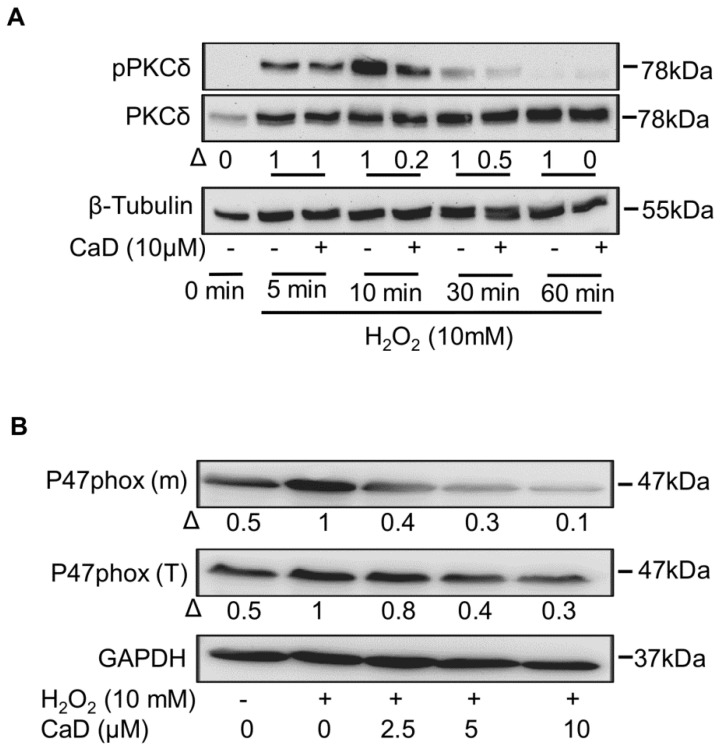
Effect of CaD on H_2_0_2_-activated PKCδ and NADPH oxidase. THP-1 monocytes were pretreated with various CaD concentrations for 1 h, followed by H_2_0_2_ (10 mmol/L) for different time points (**A**) or 24 h (**B**). Phosphorylation of PKCδ, and activation and membrane translocation of p47phox were measured by Western blotting. Δ, fold-change normalized to H_2_0_2_ only. One representative image of at least three independent experiments is depicted.

Because H_2_O_2_ activates PKCδ [50] and induces oxidative stress [51], we further investigated the effect of CaD on H_2_O_2_-induced PKCδ activation. As expected, H_2_O_2_ activated PKCδ as early as 5 min, and PKCδ phosphorylation was depleted by 30 min in H_2_O_2_-treated THP-1 cells (Figure 11A). CaD treatment significantly abolished the effect of H_2_O_2_ on PKCδ and p47phox activation (Figure 11A,B). These data showed that CaD is a ROS scavenger that downregulates PKCδ signaling.

## 4. Discussion

Calcium dobesilate (CaD) is a synthetic agent that is vasoprotective via its ability to reduce capillary permeability. Specifically, CaD is known to reduce diabetic microvascular complications, reduce oxidative stress, and inhibit inflammation. However, its mechanism(s) in mediating these events has remained unknown. Consequently, we hypothesized that CaD might reduce monocyte-to-macrophage differentiation, one specific hallmark of inflammation (and atherosclerosis) that involves signaling via PKCδ and MAPK, previously shown as mediators of CaD activity in a diabetes model [14,37].

To assess this hypothesis, we used three specific models for monocyte-to-macrophage differentiation: THP-1 leukemic monocyte treatment with (1) the phorbol ester PMA; (2) the bacterial endotoxin lipopolysaccharide (LPS); and (3) high-glucose treatment. Overall, we showed for the first time that CaD effectively affects such differentiation, as evidenced by downregulation of CD14, TLR4, and MMP9 [52]. Moreover, CaD produced consistent results in primary human macrophages. More specifically, we found that CaD inhibited CD14, TLR4, and MMP9 expression via a PKCδ/NADPH oxidase/ROS/MAPK/NF-κB-dependent pathway in PMA-treated THP-1 cells. Attenuation of this signaling cascade reduced both monocyte-to-macrophage differentiation and inflammation.

CD14 and TLR4 facilitate detection of LPS, while CD14 and TLR4 knockout mouse macrophages have impaired NF-κB pathway activation, resulting in deficient production of the pro-inflammatory cytokines IL6 and TNFα production, following LPS induction [29,30,31]. Moreover, CD14 and TLR4 play a vital role in initiating sterile inflammation related to atherosclerosis [2,3,28]. Therefore, the effect of CaD to decrease CD14 and TLR4 expression could offer one mechanism to explain why CaD inhibits NF-κB and suppresses the release of downstream NF-κB activation products—TNFα, IL-1β, IL-6, and MCP-1 in animal models of sepsis and diabetic nephropathy and retinopathy [11,13,14,32]. Indeed, in this study, CaD demonstrated potent anti-inflammatory effects by suppressing TNFα, IL-1β, and MCP-1 levels in PMA, LPS, and high glucose-treated THP-1 cells, which CaD significantly suppressed, suggesting a broad therapeutic spectrum potential of CaD [11,12,13,14,32,53].

Once we established that CaD downregulated CD14 and TLR4 expression, we differentiated THP-1 cells and primary human monocytes to macrophages in the presence of CaD and investigated their response to LPS. As expected, CaD significantly inhibited LPS-induced pro-inflammatory cytokines expression and production in both types of macrophages. These results support a novel anti-inflammatory mechanism of CaD: inferring that CaD inhibits inflammation by suppressing macrophage CD14-TLR4 expression. 

It is known that macrophages resident in human and experimental atherosclerosis co-localize with and release active matrix metalloproteinases (MMPs), including the gelatinase MMP9, which specializes in the digestion of basement membranes. Specifically, MMP9 is implicated in the pathogenesis of atherosclerosis [54], as its ablation protects apolipoprotein E-deficient mice against atherosclerosis [55]. Our observation that CaD significantly reduced MMP9 expression and activity corroborates a recent study in a rabbit model of atherosclerosis, in which CaD reduced MMP9 in local vascular walls reducing atherosclerotic plaque formation and improved endothelial function [56]. Given the importance of inflammation, macrophage activation, and MMP9 activity in atherosclerosis pathogenesis, our data suggest a promising potential of CaD as an anti-atherosclerotic agent.

To elucidate molecular mechanisms of how CaD impairs monocyte-to-macrophage differentiation and inflammation, we used a THP-1 leukemic monocyte cell model. PMA stimulates PKC-MAPK-NF-κB by acting as an analog of diacylglycerol, among the PKC isoforms, PKCα, -β, and -δ play significant roles during monocyte-to-macrophage differentiation [57,58]. In addition to PMA, LPS and high glucose activate PKCδ-MAPK-NF-κB signaling [35,36,37,38]. The NF-κB pathway, in particular, is activated downstream of MEK/ERK [59,60] and, in turn, plays a crucial role in activating inflammatory response genes. As expected, we observed that PMA, LPS, and high glucose activated PKCβII, PKCδ, and MAPK (ERK1/2, P38, and JNK); PMA stimulated PKCα expression, albeit at a low level, most likely because PKCα is lowly expressed in THP-1 cells [27]. Although CaD treatment did not affect PKCα, PKCβII, and JNK, it significantly blunted PKCδ, ERK1/2, and P38 activation. CaD, like other antioxidants, selectively inhibited PKCδ-ERK1/2-P38-dependent signaling during PMA-induced monocyte-to-macrophage differentiation [61,62]. These results correspond with previous studies in experimental diabetes where CaD inhibited PKCδ, ERK1/2, and p38 activation [11,37]. Moreover, similar to previous studies, CaD inhibited PMA-, LPS-, and high glucose-induced NF-κB activation [11,32,42]. 

Our data showed that PKCδ, MAPK, and NF-κB inhibitors significantly decreased CD14, TLR4, MMP9, and pro-inflammatory cytokines expression. It is known that knocking down PKCδ in THP-1 cells significantly decreased PMA-induced CD14 expression [46]. PKCδ plays a role in TLR4 expression, TLR4-mediated cytokine secretion [24,35,63], and MMP9 expression and activity [64]. Inhibiting or downregulating PKCδ in innate immunity cells decreases NF-κB activation and pro-inflammatory cytokines secretion [35]; inhibiting MAPK downregulates CD14, TLR4, MMP9, and pro-inflammatory markers expression [39,40,41]. Consistent with these studies, we showed that PKCδ, MAPK, and NF-κB inhibitors significantly downregulated CD14, TLR4, MMP9, and various pro-inflammatory cytokines. Therefore, through inactivating PKCδ, MAPK, and NF-κB, CaD indirectly decreased CD14, TLR4, and MMP-9 levels, inhibiting monocyte-to-macrophage differentiation and subsequent inflammation.

In addition, a PKCδ inhibitor suppressed ERK1/2, P38, and IκB-α phosphorylation to levels similar to CaD treatment, supporting previous findings that PKCδ is the important PKC isoform upstream of ERK1/2, P38, and NF-κB activation in PMA-induced THP-1 cell differentiation [46]. From these results, it is clear that PKCδ mediated the effect of CaD during monocyte-to-macrophage differentiation and inflammation via inhibiting MAPK and NF-κB activation. 

As oxidative stress is well established as associated with inflammation, we examined CaD effects on ROS. Specifically, NADPH oxidase is accepted as the most important inducer of phagocytic cell generation of ROS (especially H_2_O_2_), which acts as a second messenger in activating signaling pathways that induce monocyte adhesion, invasion, and migration in atherosclerosis [65]. ROS is also implicated in various cell signaling processes, including monocyte-to-macrophage differentiation [43], via glucose or PMA activation of PKC/MAPK signaling [66]; this is suppressed by antioxidants, such as curcumin, NAC, vitamin E, and vitamin C [39,67]. Additionally, CaD is a potent antioxidant in both in vitro and in vivo animal models [11,12,68,69], although its exact mechanism is not fully elucidated. In the present study, we found that the ROS scavenger NAC and the NADPH oxidase inhibitor DPI inhibited CD14, TLR4, and MMP-9 expression in PMA-treated cells, while also demonstrating that CaD markedly suppressed ROS production during PMA-induced THP-1 differentiation. This is the first report of ROS modulating CD14 expression in human macrophages to the best of our knowledge. Recently, Akhter et al. reported that treatment of peripheral blood mononuclear cells with H_2_O_2_ induced TLR4 expression via ROS production [51], which also upregulates macrophage MMP-9 expression and cell migration [70]. These findings clearly show that CaD modulated monocyte-to-macrophage differentiation and inflammation via ROS scavenging. 

The severity of atherosclerosis is well associated with *NOX2* upregulation, which increases intracellular oxidative stress in macrophages [65]. Additionally, upregulated *NOX2* correlates with plaque macrophage content in human coronary atherosclerosis [71]. In various disease models, PMA, LPS, and high glucose are known to activate NADPH oxidase assembly by promoting the phosphorylation and translocation of cytosolic p47phox [72,73,74,75]. This study demonstrated a novel antioxidant mechanism in which CaD downregulates *NOX2* expression while also attenuating p47phox membrane translocation during THP-1 stimulation with PMA, LPS, and high glucose. Overall, these findings agree well with previous studies where antioxidants inhibited monocyte-to-macrophage differentiation and associated inflammation [73,76]. These results showed that CaD could block PMA, LPS, and glucose-induced ROS generation through the inhibition of p47phox/NOX2 activation. Thus, CaD could potentially provide a novel therapeutic approach targeting NOX2/p47phox-mediated complications, such as atherosclerosis [77], sepsis [78], and diabetic nephropathy [79]. 

Since PKCδ regulates p47phox activation and translocation (thus contributing to monocyte NADPH oxidase activity [80]), we found both events to be significantly inhibited by rottlerin, in line with a previous study [80]. This suggests that NOX2/p47phox is a downstream target of PKCδ, indicating a mechanism for NOX2/p47phox signal inhibition by CaD.

Our findings that CaD regulated PKCδ activation and no known CaD- PKCδ interactions prompted us to investigate how CaD regulates PKCδ during monocyte-to-macrophage differentiation. During oxidative stress, ROS can act both downstream and upstream of PKC. For example, ROS provides signal amplification during PMA-, LPS-, and high glucose-induced monocyte-to-macrophage differentiation or activation. This establishes a positive feedback loop to sustain ROS-PKCδ signaling [38,44], as ROS is also an upstream regulator of PKC, under high glucose, in human peritoneal mesothelial cells [81] and antioxidants suppressed PKC activity in phorbol ester-stimulated human hepatoma cells [82]. Consistent with a previous report [83], the ROS scavenger NAC significantly inhibited PKCδ activation and expression, likewise indicating that ROS act upstream of PKCδ. 

Indeed, H_2_O_2_ treatment activated PKCδ phosphorylation and p47phox activation, which was abrogated considerably by CaD treatment, proving CaD to be a ROS scavenger that downregulates PKCδ signaling. Our data clearly showed that during monocyte-to-macrophage differentiation and inflammation, CaD inhibits PKCδ, NADPH oxidase, ROS, MAPK, and NF-κB activation, decreasing the expression of CD14, TLR4, MMP9, and pro-inflammatory cytokines. Several studies support the hypothesis that a PKC isoform inhibitor (such as PKCδ inhibitor) might prevent or decrease hyperglycemia-induced atherosclerosis [84]. Therefore, our results further suggest that CaD’s ability to suppress PKCδ activation may likewise provide advantages in modulating the process of atherosclerosis. 

One limitation of this study is that we cannot rule out that CaD downregulates the PKCδ pathway via VEGF signaling since CaD is also a VEGF signaling inhibitor in endothelial cells [14]. ROS scavengers can also inhibit angiogenic factor production due to a direct effect on monocytes/macrophages [85], and antioxidants, such as phenolic compounds, vitamins C and E, likewise inhibit VEGF-VEGFR signaling [86,87]. Accordingly, in this study, we detected a significant decrease in VEGF expression in THP-1 cells treated with PMA in the presence of CaD (Supplementary Figure S7). Nonetheless, this is an exciting topic for future research.

In summary, we (1) demonstrated for the first time that CaD effectively downregulates CD14, TLR4, MMP9, IL1β, TNFα, and MCP-1 expression during THP-1 macrophage differentiation and translated our findings to primary human macrophages; (2) provided evidence that CaD inhibits PKCδ-dependent NADPH oxidase-MAPK-and NF-κB signaling, and (3) illustrated that CaD downregulates PKCδ activation by inhibiting ROS-driven oxidative stress.

## 5. Conclusions

The present study identified a novel mechanism of how CaD alleviates metabolic and infectious inflammation, including suppressed oxidative stress-dependent CD14/TLR4 expression and the associated inflammation. The results suggest that CaD can prevent or treat inflammatory conditions related to inflammatory macrophages. Future research should further develop and confirm these findings in in vivo models.

## Data Availability

All the data presented in this study are included in the article and its Supplementary Material.

## Supplementary Materials

The following are available online at https://www.mdpi.com/article/10.3390/antiox10111798/s1, Figure S1: Effect of calcium dobesilate (CaD) onTHP-1 cells viability and adhesion, Figure S2: Effect of calcium dobesilate (CaD) on LPS stimulated THP-1 monocytes, Figure S3: Effect of calcium dobesilate (caD) on high glucose-induced inflammation, Figure S4: The effect of calcium dobesilate (CaD) on signaling pathways induced by high glucose and LPS, Figure S5: N-acetylcysteine (NAC) inhibits monocyte-to-macrophage differentiation and inflammation, Figure S6: PKCδ is the proximal target of calcium dobesilate (CaD), Figure S7: Calcium dobesilate (CaD) downregulates VEGF expression.
