# Supplementary material for: Calcium Dobesilate Modulates PKCδ-NADPH Oxidase- MAPK-NF-κB Signaling Pathway to Reduce CD14, TLR4, and MMP9 Expression during Monocyte-to-Macrophage Differentiation: Potential Therapeutic Implications for Atherosclerosis
Source: Antioxidants (Basel). 2021 Nov 11;10(11):1798. doi: 10.3390/antiox10111798 (PMC8615002; doi:10.3390/antiox10111798)
Supplement: Supplementary file 1 [file antioxidants-10-01798-s001.zip › antioxidants-1461701-supplementary.pdf]

A

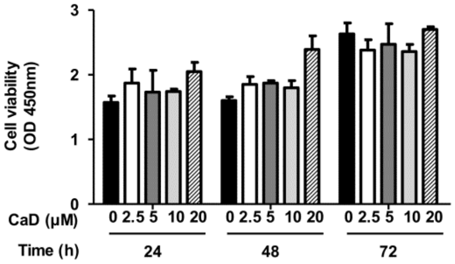

B

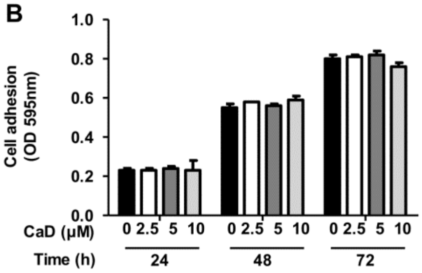

C

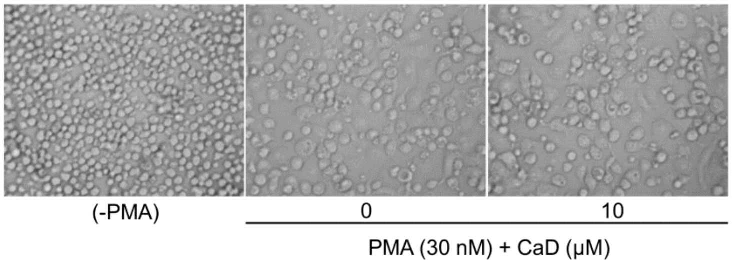

Supplementary Figure S1. Effect of Calcium dobesilate (CaD) onTHP-1 cells viability and adhesion. THP-1 monocytes were pre-treated with different concentrations of CaD for various time points, and the effect of CaD on cellular viability was measured using CCK-8 kit (A). THP-1 cells were treated as in A for 1 h, followed by stimulation with PMA (30 nmol/L) for various time points. Cellular adhesion was measured using the crystal violet staining method (B) and a bright-field microscope after 48 h (C).

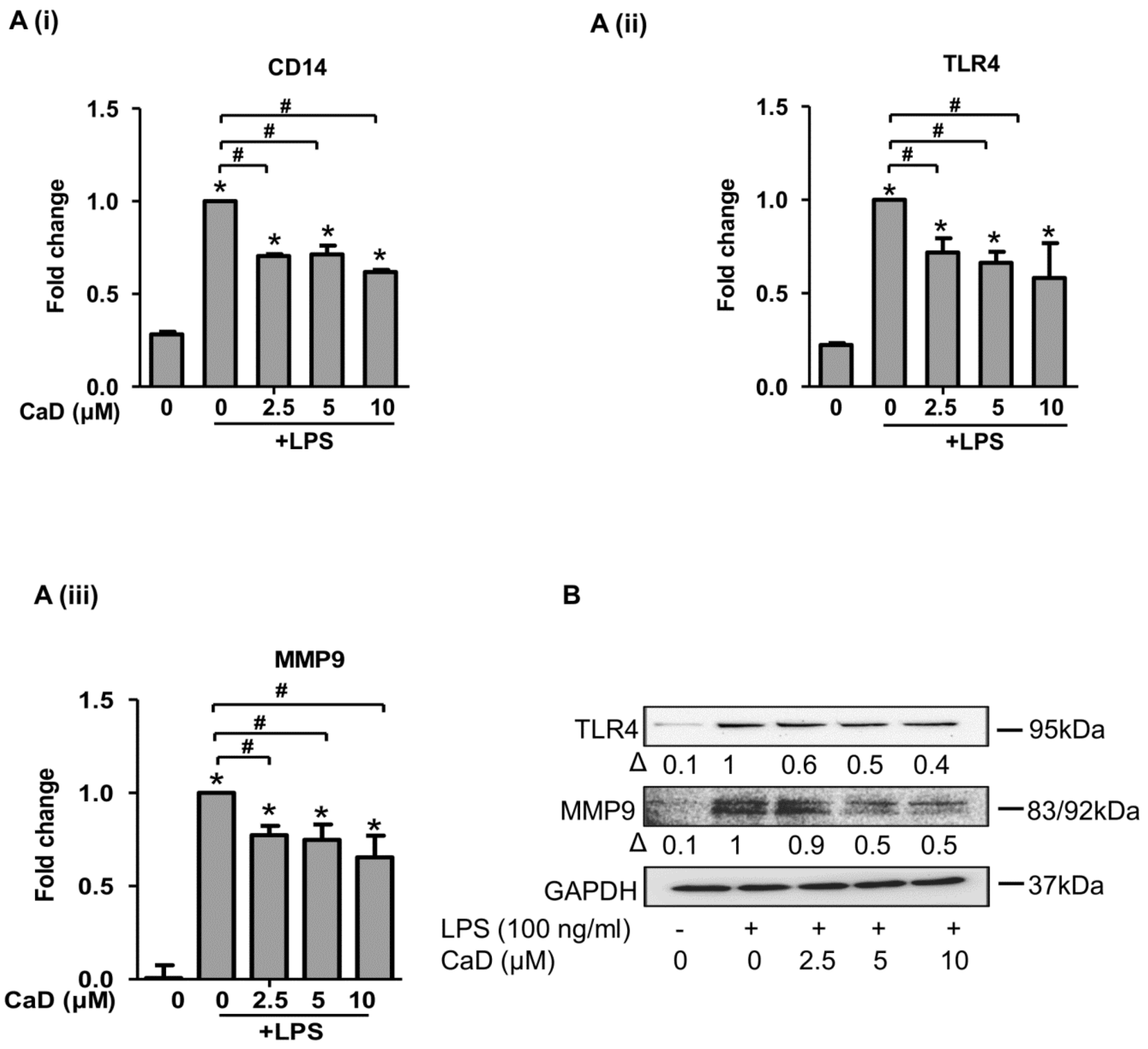

Supplementary Figure S2. Effect of CaD on LPS stimulated THP-1 monocytes. THP-1 monocytes were pre-treated with different concentrations of CaD as indicated for 1 h, followed by stimulation with LPS (100 ng/ml) for 24 h. Expression of differentiation and inflammation marker transcripts (A) and protein (B) levels were measured as already described. Δ, fold-change normalized to LPS only (n = 3, mean ± SEM. *$P < 0.05$ vs. no LPS, #$P < 0.05$ vs. LPS only, one-way ANOVA). Western blots represent one from at least three independent experiments.

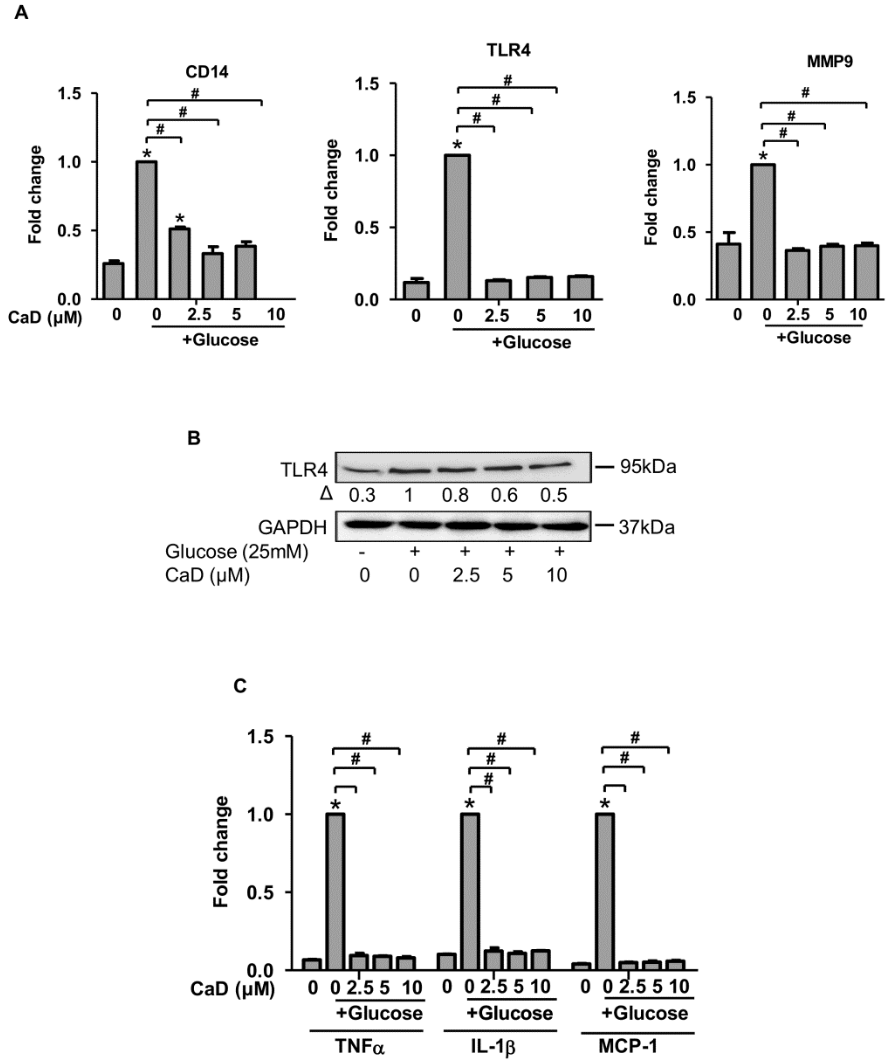

Supplementary Figure S3. Effect of CaD on high glucose-induced inflammation. THP-1 monocytes were pre-treated with different concentrations of CaD as indicated for 1 h and then treated with high glucose (25 mmol/L) for 48 h. Expression of differentiation and inflammation marker transcripts (A and C) and protein (B) levels were measured as already described. Δ, fold-change normalized to glucose only (n = 3, mean ± SEM. *$P < 0.05$ vs. no glucose, #$P < 0.05$ vs. glucose only, one-way ANOVA). Western blots represent one from at least three independent experiments.

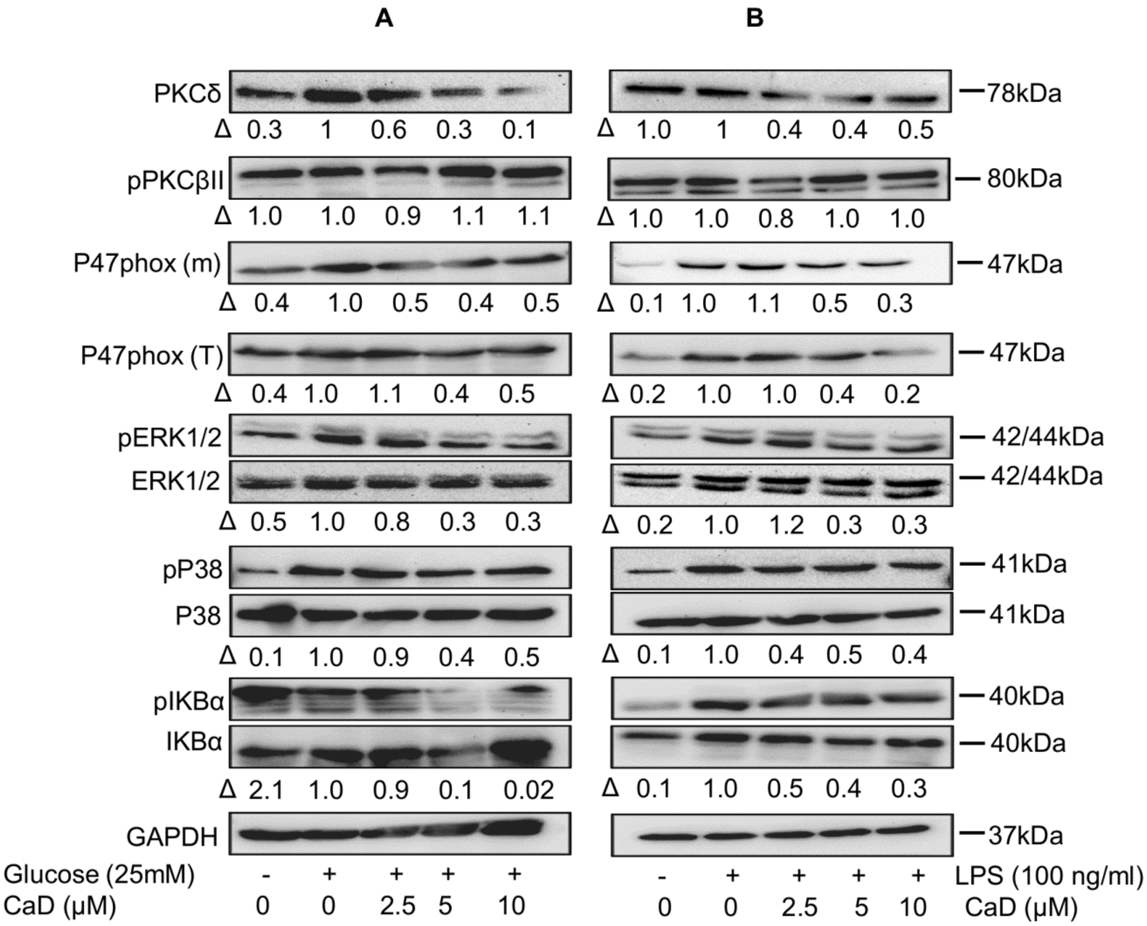

Supplementary Figure S4. The effect of CaD on signaling pathways induced by high glucose and LPS. THP-1 monocytes were pre-treated with different concentrations of CaD as indicated for 1 h and then treated with either high glucose (25 mmol/L) for 48 h (A) or LPS (100 ng/ml) for 30 minutes (B). Activation PKC-MAPK-NF-κB) was measured by Western blotting. Δ, fold-change normalized to LPS or glucose only. One representative of at least three independent experiments is depicted.

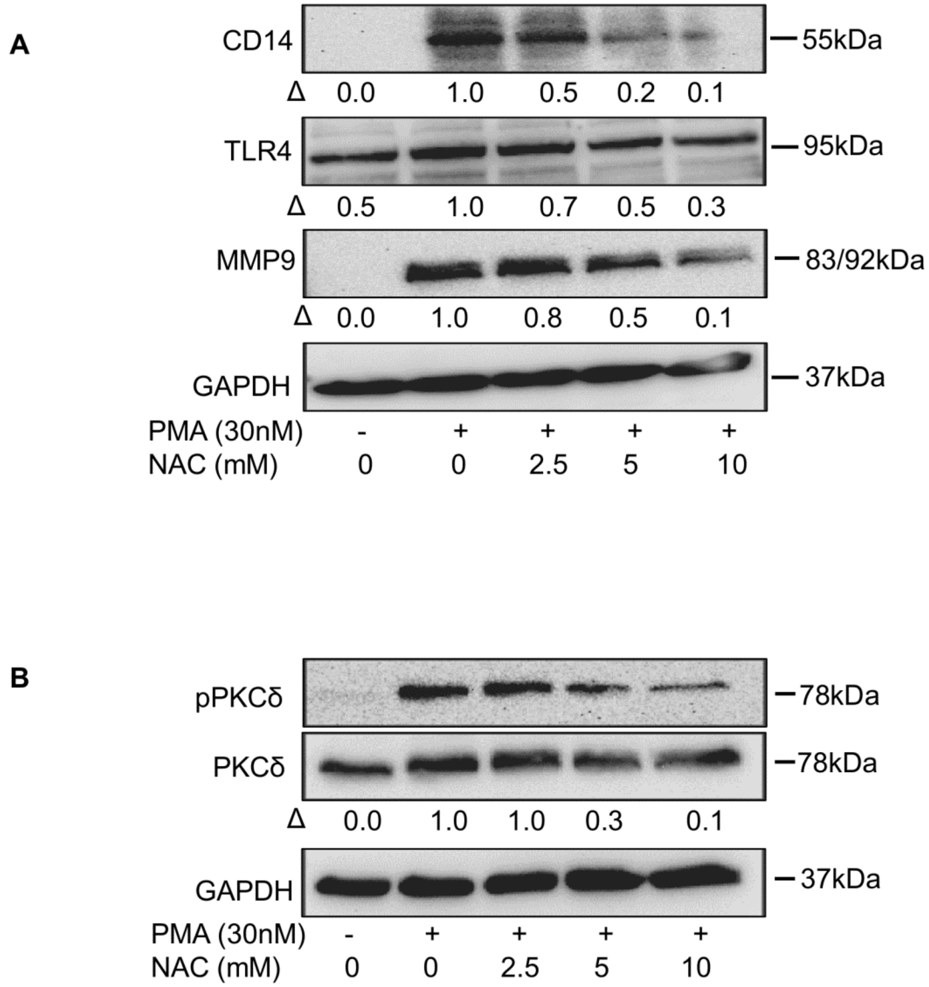

Supplementary Figure S5. N-acetylcysteine (NAC) inhibits monocyte-to-macrophage differentiation and inflammation. THP-1 monocytes were pre-treated with various concentrations of NAC for 1 h, followed by stimulation with PMA (30 nmol/L) for 72 h (A), or 30 minutes (B). Expression of differentiation/inflammatory markers (A) and phosphorylation of PKCδ (B) were measured by Western blotting. Δ, fold-change normalized to PMA only. One of at least three independent experiments is depicted.

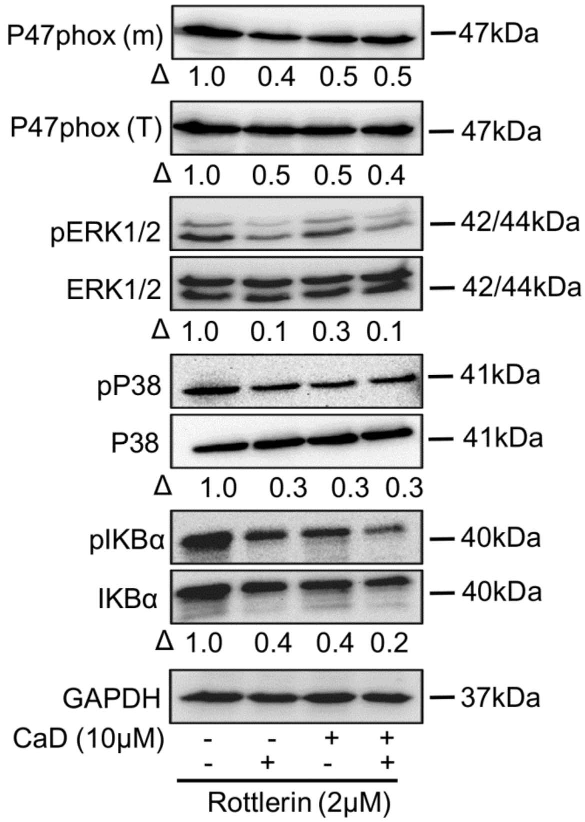

Supplementary Figure S6. PKCδ is the proximal target of CaD. THP-1 monocytes were pre-treated with rottlerin for 1 h, followed by CaD for 1h, then stimulated with PMA (30 nmol/L) for 30 minutes. Phosphorylation of MAPK and IKBα; total protein expression (T) and membrane translocation (m) of p47phox was measured by Western blotting. Δ, fold-change normalized to PMA only. One of at least three independent experiments is depicted.

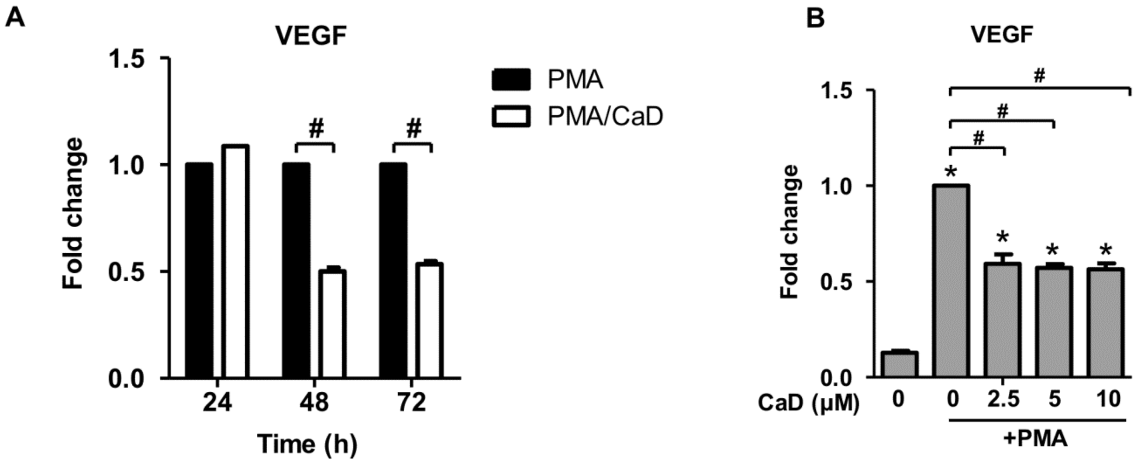

Supplementary Figure S7. CaD down-regulates VEGF expression. THP-1 monocytes were pre-treated with (10 μmol/L) CaD for one h followed by stimulation with PMA (30 nmol/L) for various time points (A), or THP-1 monocytes were treated with various concentrations (0–10 μmol/L) of CaD for 1 h followed by PMA treatment for 48 h (B). Expression of VEGF was measured by quantitative RT-PCR [n = 3, mean ± SEM. *P < 0.05 vs. no treatment, #P < 0.05 vs. PMA only, Student t-test (A), or one-way ANOVA (B)].
